# Coarse-grained modelling of the structural properties of DNA origami

**DOI:** 10.1093/nar/gky1304

**Published:** 2019-01-03

**Authors:** Benedict E K Snodin, John S Schreck, Flavio Romano, Ard A Louis, Jonathan P K Doye

**Affiliations:** 1 Physical, and Theoretical Chemistry Laboratory, Department of Chemistry, South Parks Road, Oxford OX1 3QZ, UK; 2 Department of Chemical Engineering, Columbia University, 500 W 120th Street, New York, NY 10027, USA; 3 Dipartimento di Scienze Molecolari e Nanosistemi, Universit Ca’ Foscari, Via Torino 155, 30172 Venezia Mestre, Italy; 4 Rudolf Peierls Centre for Theoretical Physics, University of Oxford, 1 Keble Road, Oxford OX1 3NP, UK

## Abstract

We use the oxDNA coarse-grained model to provide a detailed characterization of the fundamental structural properties of DNA origami, focussing on archetypal 2D and 3D origami. The model reproduces well the characteristic pattern of helix bending in a 2D origami, showing that it stems from the intrinsic tendency of anti-parallel four-way junctions to splay apart, a tendency that is enhanced both by less screened electrostatic interactions and by increased thermal motion. We also compare to the structure of a 3D origami whose structure has been determined by cryo-electron microscopy. The oxDNA average structure has a root-mean-square deviation from the experimental structure of 8.4 Å, which is of the order of the experimental resolution. These results illustrate that the oxDNA model is capable of providing detailed and accurate insights into the structure of DNA origami, and has the potential to be used to routinely pre-screen putative origami designs and to investigate the molecular mechanisms that regulate the properties of DNA origami.

## INTRODUCTION

DNA nanotechnology seeks to use the specificity of the Watson–Crick base pairing and the programmability possible through the DNA sequence to design self-assembling nanoscale DNA structures and devices. The most prevalent technique used is probably that of DNA origami in which a long viral ‘scaffold’ DNA single strand is folded up into virtually any arbitrary structure by the addition of many different ‘staple’ strands that bind to multiple specific domains on the scaffold (1,2). The initial designs were two-dimensional (3) but were soon generalized to three-dimensional shapes (4), and then to bent, twisted (5) and curved (6) structures through the introduction of internal mechanical stresses. The increasing usage of DNA origami was particularly facilitated by the development of computer-aided design tools, such as caDNAno (7). These original approaches produced structures involving mainly bundles of locally parallel double helices held together by four-way junctions. More recently, scaffolded origami approaches have been developed that generate more open ‘wireframe’ structures (8–11), aided by the vHelix (8) and DAEDALUS (9) design programmes. The structural control and the addressability provided by the DNA origami technique naturally have led to many types of applications (12), particularly in the areas of biosensing (13), drug delivery (14,15) and nanofabrication (16,17).

In Rothemund’s original paper, the structures of the origami were characterized by atomic force microscopy (AFM). The images were used to confirm that the origami had folded into the designed structures without significant defects, and identified structural features of the origami, such as what we here term the ‘weave’ pattern where the helices, rather than being straight, splay out between four-way junctions, thus leading to the characteristic pattern where the helices weave back and forth between adjacent helices (3). Such microscopy studies (by AFM and transmission electron microscopy) are probably the most prevalent way of characterizing the structures of DNA origami, but are usually limited in terms of the fine-grained detail that can be obtained. Furthermore, adsorption onto a surface may perturb the structure, especially for 2D origami, which may be flattened and made to look more ordered because of the suppression of out-of-plane thermal fluctuations.

Solutions-based measurements can be performed by, for example, small-angle X-ray scattering (SAXS) and FRET, but SAXS interpretation usually requires a structural model (and its computed SAXS pattern) for comparison (18–21). FRET can potentially provide detailed measurements of selected distances, but has been relatively little used to provide detailed structural analysis of origami objects (22,23).

Cryo-EM can potentially provide the most detailed structural analysis. For example, Bai *et al.* were able to obtain a high-resolution structure for a three-dimensional origami where an all-atom structure was fitted to the obtained electron density maps (24). However, such detailed studies are unlikely to be a routine approach. More commonly, cryoEM has been used at a somewhat lower level of resolution, particularly for polyhedral nanostructures (9,25,26). Very recently, particle electron tomography has also begun to be applied allowing visualization of the 3D structure of individual DNA nanostructures (27,28).

Given both the difficulty of obtaining high-resolution structural information and the potential utility of being able to predict structural properties prior to experimental realization, computational modelling of the structure of DNA origami has the potential to play a significant role in the field (29). All-atom simulations have the potential to provide the most detailed structural insights (30–35). Notably, the Aksimentiev group have simulated a number of origami nanostructures (30,32–34), including even an origami nanopore inserted into a membrane (33). However, such simulations are extremely computationally intensive and cannot be performed routinely. Furthermore, even for the relatively stiff origami considered in these studies, it is not clear that they have fully equilibrated on the simulation time scales (34). More promising as a general tool is an approach where only the atoms of the origami (but not the water environment) are simulated and an elastic network is used to constrain the origami in its assembled state; these constraints are applied to the base pairing and base stacking interactions, and also to the distance between neighboring helices (34).

A computationally less expensive approach is to use coarse-grained models in which the basic units are no longer atoms, but some larger moiety, be it a nucleotide (36–38), a base pair (39,40) or a section of a double helix (41–46). Such approaches of course inevitably lead to a lower level of structural detail, and the accuracy of their properties will depend on the quality of the parameterization.

By far the most widely-used approach is CanDo as it allows efficient and reliable structural screening of potential origami designs through a simple-to-use web interface (41–44). However, its lack of excluded volume interaction means that it may not be appropriate for flexible origami whose structures are not fully mechanically constrained. Furthermore, as with any model whose basic unit is above the level of a nucleotide, there is no coupling to intra-base-pair degrees of freedom; consequently processes such as duplex fraying, junction migration, and breaking of base pairs due to internal stresses cannot be resolved. Finally, it has a simplified representation of single-stranded DNA, and so cannot take into account, for example, secondary structure formation.

All these potential deficiencies can be addressed by a nucleotide-level model, albeit at greater computational expense. Although there are a number of such models at this level of detail (47–49), here we explore in detail the description of DNA origami nanostructures provided by the oxDNA model (36–38). This model has been particularly successful at describing a wide variety of biophysical properties of DNA (36,38,50–54), and has been applied to a significant number of DNA nanotechnology systems (38,55–70).

What are the features that make the oxDNA model particularly appropriate to study DNA origami? Firstly, it is able to accurately reproduce DNA’s basic structural properties. Properties such as the DNA pitch are particularly important, as the large size of DNA origami means that small deviations can lead to internal stresses that lead to global twisting of the origami—note that in the second version of the oxDNA model the duplex pitch and the twist at nicks and junctions were fine-tuned to correct just such an issue (38). Secondly, it is able to capture the mechanical properties of DNA such as the persistence length and torsional modulus (36,38,52,54); these are important for correctly capturing both the thermal fluctuations of DNA origami and the equilibrium structure when internal stresses are deliberately designed into the origami to cause overall bend and twist (5). For example, oxDNA can capture the global right- and left-handed twisting when insertions or deletions, respectively, are introduced into an untwisted 3D origami design (38). Thirdly, it has a very good representation of the thermodynamics of hybridization (36–38) allowing it to capture fraying, the breaking of base pairs due to more extreme internal or external stresses, and secondary structure formation in single strands. Fourthly, it has a good representation of the mechanical properties of single-stranded DNA (37); this is relevant to that subset of origami that use ssDNA to introduce flexibility (71), exert forces (72) or brace tensegrity structures (73).

OxDNA is also able to naturally capture the mechanical behaviour of other sites. For example, in unstressed DNA it is generally favourable for the DNA to stack across a nick and in this state it has very similar elastic properties to standard duplex DNA. However, for a relatively small free-energy cost this stacking can be broken and the two halves of the duplex can then rotate relatively freely about the hinge point (53). Lower-resolution models are typically unable to capture the two-state character associated with the nick and instead associate a single set of moduli with the nick.

OxDNA has previously been used to characterize a number of specific DNA origami nanostructures with good success (38,63–65,68,69). Here, our aim is to provide a detailed structural analysis of some of the basic features of DNA origami and to test the reliability of the oxDNA model by comparing to the most structurally detailed available experimental data. The systems that we will study are an archetypal 2D origami tile that has recently been characterized by SAXS (21), and the 3D origami of (24). To better understand the origins of some of the structural features, we also characterize the free-energy landscape of an unconstrained four-way junction, a key motif in DNA origami.

## MATERIALS AND METHODS

In this work, we have used the version of the oxDNA model described in (38) (sometimes called ‘oxDNA2’) for which the properties have been fine-tuned to capture origami twist. This version of the model also has an explicit dependence on the ionic strength through a Debye–Hückel-like term in the potential. Note, such a simple form is, of course, not capable of capturing ion-specific effects. This electrostatic term was parameterized so that the dependence of duplex melting on [Na^+^] is reproduced. However, oxDNA overestimates the stability of the stacked form of the Holliday junction as a function of [Na^+^]. As it happens, this is an advantage when modelling DNA origami, as in experiments origami are typically assembled in a buffer that contains Mg^2+^, which is known to stabilize the stacked form of the Holliday junction in an ion-specific manner (74). Thus, [Na^+^] = 0.5 M, the solution conditions at which we chose to model the origami using oxDNA, is a reasonable choice to mimic the experimental solution conditions, with only very small changes in the structural properties of the oxDNA origami occurring as the concentration is further increased. By contrast, extremely high values of [Na^+^] have to be used in experiment to induce origami assembly (75) probably because of the relative instability of the stacked Holliday junction in [Na^+^] solutions (74). We also note that, we used the oxDNA model with average-strength (rather than sequence-dependent) interactions, as we are interested in generic structural properties.

To generate initial origami structures, we have developed a publicly available script to turn a caDNAno file into a starting oxDNA configuration. The initial structures generated may locally be subject to very large forces due to overlaps or somewhat extended backbones. As such large forces are potentially problematic for a simulation, we have a developed an approach to first relax configurations prior to them being simulated (see Supplementary Data for details).

The molecular dynamics simulations use an Andersen-like thermostat, in which the velocities and angular velocities are periodically refreshed from a Maxwell-Boltzmann distribution, to generate diffusive motion of the nucleotides in the absence of explicit solvent (76). To aid the simulation of large origami, we use a GPU-enabled verion of our simulation code (77).

## RESULTS AND DISCUSSION

### Holliday junction structure

Before we directly address origami structure, we first consider the properties of a single four-way junction, also known as a Holliday junction, as they are an essential feature of origami designs. These occur wherever two strands, usually staple strands, cross from one double helix to another within the origami. Thus the junctions play the vital role of joining adjacent double helices together. Because each origami contains many such junctions, their structural properties can potentially have a major effect on the structure of the origami.

The structure of a single isolated Holliday junction has been characterised experimentally (74,78) through X-ray crystallography (79,80), AFM (81) and FRET measurements (82–84). Depending on the experimental conditions, a Holliday junction can exist in an open or stacked conformation, with the open conformation favoured at low concentrations of metal ions, and with divalent ions being particularly effective at stabilizing the stacked conformation (78). Because the adoption of a stacked geometry is important to the structural integrity of standard DNA origami, ‘high salt’ conditions are used in origami experiments, normally, but not necessarily (75), in a solution containing significant Mg^2+^. For this reason, here we only consider the stacked conformation of the junction, this form being favoured by oxDNA for the salt conditions we consider here (0.5 M).

**Figure 1.**
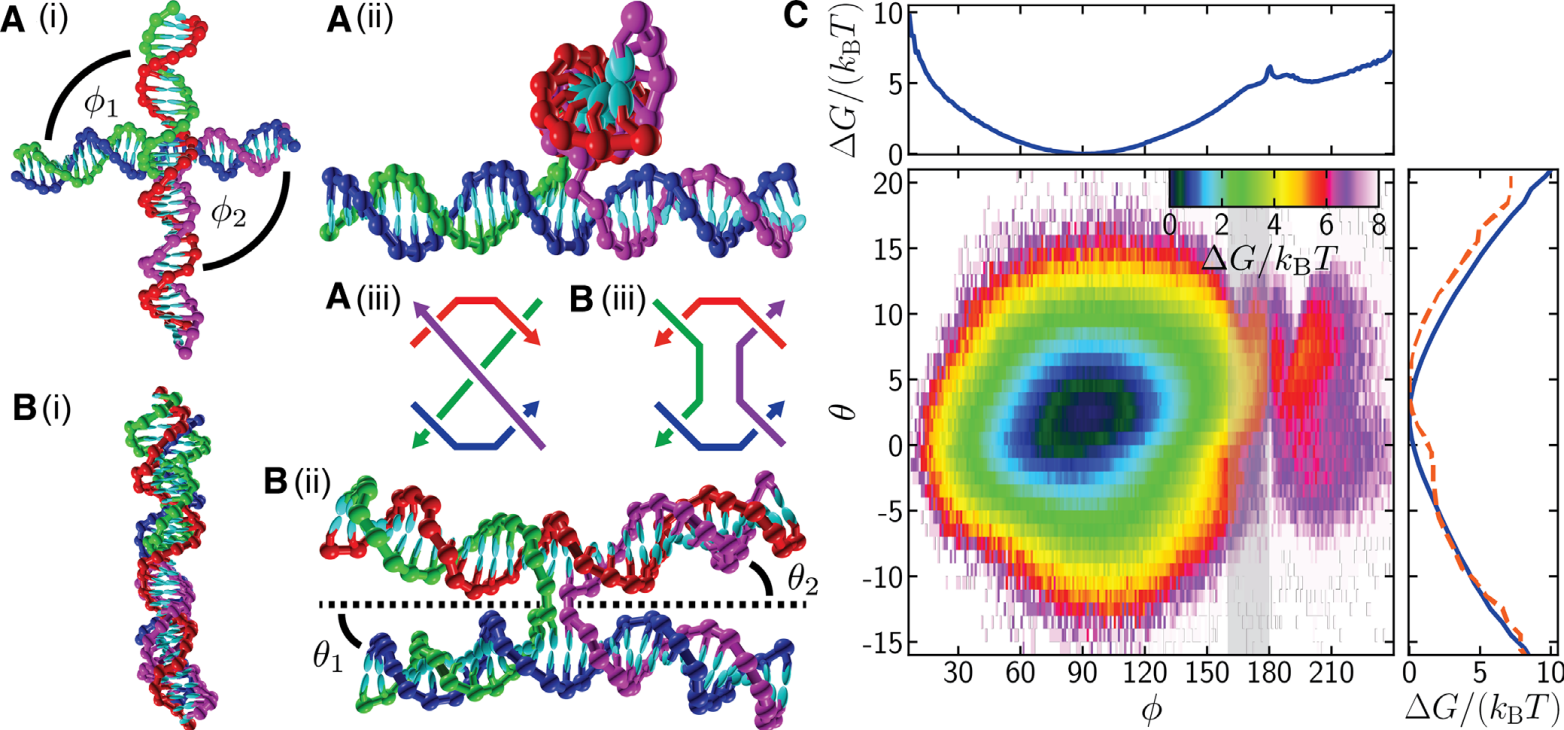
(A) and (B) OxDNA configurations for a single Holliday junction at 0.5 M salt that are representative of (**A**) the left-handed global free-energy minimum (ϕ = 95.5°, θ = 2.5°) and (**B**) an anti-parallel junction (ϕ = 180°, θ = 5°). (i) and (ii) provide perpendicular views of the configurations. In both cases the green and purple strands cross from one double helix to another, while the blue and red strands carry straight on along their double helix. (A)(iii) and (B)(iii) provide schematics of a left-handed parallel and an anti-parallel junction, respectively. (**C**) The Holliday-junction free-energy landscape as a function of ϕ and θ. One-dimensional free-energy profiles for ϕ and θ are also shown. The full free-energy profile for θ is shown as a solid blue line, while the dashed red line is for the subregion 160° ≤ ϕ ≤ 180°, shaded grey in the free-energy landscape. ϕ = (ϕ_1_ + ϕ_2_)/2 is a measure of the chiral twist of the junction, where ϕ_1_ and ϕ_2_ are the angles between the arms as indicated in (A)(i). θ = (θ_1_ + θ_2_)/2 is the average angle between each arm and the plane of the junction, as indicated in (B)(ii). The plane of the junction, represented by the dotted line in (B)(ii), is perpendicular to the plane of the paper and passes through the midpoints of the exchanging strands. The temperature used was 296.15 K.

In order to quantify the structure of the junction, we define two angles: ϕ = (ϕ_1_ + ϕ_2_)/2, which measures the average twist angle between pairs of arms; and θ = (θ_1_ + θ_2_)/2, which measures the average angle between the arms and the plane of the junction (Figure 1). ϕ is defined such that ϕ = 0° for a parallel junction and ϕ = 180° for an anti-parallel junction, where the (anti-)parallel character refers to the relative orientation of the two strands that do not cross in the stacked junction. The junctions in origami are typically anti-parallel. A junction is said to be right-handed if ϕ > 180° and left-handed if ϕ < 180°. θ provides a measure of how much the DNA helices bend at the junction and will be particularly relevant when we consider the ‘weave’ pattern for DNA origami. Precise definitions of the angles are given in the Supplementary Data.

To characterize the free-energy landscape of the junction, we ran molecular dynamics simulations of an isolated Holliday junction with arms that are 16 base pairs long. We used a biasing potential (defined in the Supplementary Data) to window the simulations in ϕ, with the particular aim of accelerating the sampling for ϕ values near 180°, as this is the region relevant to the junctions in origami. The sequence was chosen to prevent branch migration. The junction can adopt one of two stacked isomers. In order to simplify the analysis, we only considered one of the isomers and so configurations that were determined to be in the wrong isomer were discarded. Further details regarding the simulations, including the DNA strand sequences and how a configuration’s isomeric state is determined, are given in the Supplementary Data.

The resulting free-energy landscape is depicted in Figure 1C. The free-energy minimum for the junction is at (ϕ, θ) = (95.5°, 2.5°), while integrating over θ gives a preferred ϕ angle of 90.5°, with a mean value of 92.0°. Thus, Holliday junctions prefer to be left-handed in the oxDNA model. However, the junctions observed in crystal structures are usually right-handed with ϕ ≈ 240° (78). The right-handed character of stacked Holliday junctions in solution is also confirmed by FRET (84). The preferred value of ϕ that we see with oxDNA is simply that required to align the backbone sites of the two double helices at the junction. In this way, the distance between the two double helices can be maximized, reducing the steric and electrostatic repulsion between them. At other angles the bonds between the two helices are twisted, bringing the helices closer together at the junction.

The cause of this difference in the preferred geometry is probably the model’s simplified representation of the backbone and its associated excluded volume. However, we note that we know of no coarse-grained DNA model for which a right-handed junction naturally emerges from the model. For example, the next most widely-used nucleotide-level coarse-grained model also exhibits left-handed junctions (85). Furthermore, we also note that a crystallized left-handed junction has been reported (86) for an RNA-DNA complex, and that both chiral forms have been seen as local minima for a junction in solution in all-atom simulations (87).

That oxDNA is unable to reproduce the experimental junction crystal structures’ preference to be right-handed is, fortunately, not particularly detrimental to modelling origami structure with oxDNA for the following reasons. Firstly, the helices are able to rotate relatively freely about the crossover point and so the isolated junction is relatively flexible in ϕ (although clearly the junction will very rarely adopt a configuration with ϕ ∼ 240°). Secondly, in an origami the junctions are constrained to ϕ values close to 180°, i.e. 90° from the preferred junction angle for oxDNA and 60° for real DNA, so the junctions in both cases would be expected to have a somewhat similar level of stress, albeit with a torque in the opposite direction. Indeed, recent oxDNA results on a 2D DNA brick system have shown that oxDNA can reproduce the melting point of such systems very well (to within 2°C of experiment) without any adjustment to the model (66). This would suggest that oxDNA can capture well the thermodynamic cost of the anti-parallel junctions in these DNA brick systems, albeit noting that the junctions are less restricted than those considered here as they involve only one strand crossing.

Finally, junctions that have been experimentally resolved within a 3D origami have been found to exhibit ϕ angles slightly below 180° (24), a somewhat counter-intuitive result because one would perhaps expect deviations to take the junction towards (not away from) the preferred geometry. However, this is beneficial for oxDNA modelling of origami, because, as we will see, the origami junctions in oxDNA do somewhat twist towards their preferred left-handed orientation. This tendency to have a slight left-handed twist has also been observed in atomistic simulations of 3D origami (30). Although the cause of this left-handed twist in the experimental case is not obvious, it must reflect both the details of the real free-energy landscape (as a function of ϕ) and the constraints placed on the junctions within the origami. For oxDNA, there is a maximum at ϕ ∼ 180°, caused by the larger repulsions when the two helices are aligned, but with a greater slope away from the maximum towards the preferred left-handed configurations.

The free-energy profile for θ in Figure 1(c) shows a slight preference for a positive θ, with a free-energy minimum at θ = 2.5°, corresponding to a tendency for the helix arms to bend slightly away from the plane of the junction. The effect is greater, with the minimum at θ = 4.5°, for a subset of the data for which 160° ≤ ϕ ≤ 180°, the region likely to be relevant within origami. A simple argument explaining this behaviour is that negative values of θ will cause the arms to bump into each other more often, and this becomes more likely when the arms are approximately aligned, as for ϕ ≈ 180°. Furthermore, the twisting of the inter-helix bonds for anti-parallel junctions will lead to increased repulsion local to the junction.

Intriguingly, however, this argument would lead us to expect a similar effect as we move towards ϕ ≈ 0°, but this effect is not evident from the free-energy landscape; instead the landscape becomes more symmetric about θ = 0 in this region. Interestingly, the free-energy cost of forming a parallel junction is also much more than that of an anti-parallel junction. This may help to explain why DNA nanostructures with parallel junctions that reliably assemble have generally been more difficult to design (88,89). As ϕ ≈ 0° configurations are not relevant to junctions within conventional DNA origami we do not investigate the origins of these effects further.

### Structural properties of a 2D origami

The coupling between many Holliday junctions present in a DNA origami generates a rich set of structural properties. We first consider 2D origami, which consist of a single ‘sheet’ of (anti-)parallel DNA helices joined by crossovers. The particular design on which we focus (21) has a very regular pattern of crossovers and staples (see Supplementary Figure S2 for the caDNAno representation of the structure). Rothemund’s original 2D origami tiles have been shown to be somewhat (right) twisted (90) (a feature oxDNA reproduces (65)), because of a slight mismatch between the pitch of DNA (∼10.5 bp) and the separation between the junctions (32 bp for three helical turns). The current design has included a suitable number of sections with 31 base pairs between equivalent junctions in order to remove this net twist.

**Figure 2.**
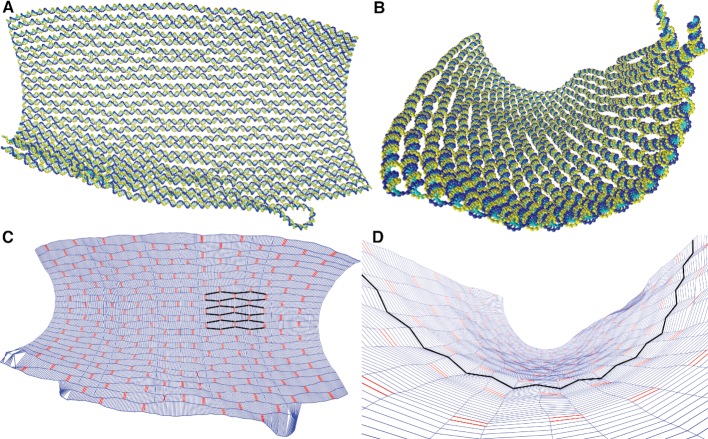
(A, B) Different representations and viewpoints of the average structure of the 2D origami. (**C**) and (**D**) provide a ‘chickenwire’ representation of the tile. In (C) the tile is shown from the front so that the weave pattern can be clearly seen (as highlighted by helix axes in black); and (D) at an angle to show the corrugation pattern on the tile’s surface (as highlighted by the inter-helix vectors in black). In (C) lines running horizontally along the origami show the axes of the double helices that make up the origami; pairs of red vertical lines represent double crossovers, while blue vertical lines represent the inter-helix vectors used for the quantitative analysis of the weave and corrugation. Deviations from the typical structure, such as that seen in the bottom left corner of the origami, are caused by staple melting or branch migration.

The average structure of the origami is shown in Figure 2 (see Supplementary Data for details of how the average structure was computed). The origami sheet is not noticeably twisted, but there is some modest curvature (as also predicted by CanDo (41)). SAXS experiments on this tile by Baker *et al.* are consistent with a flat to moderately curved shape (21). Although the structure does fluctuate considerably, here we focus on the average structure. Note that, when adsorbed onto a surface (as is the case for most experimental structural studies of origami), rather than in solution, we would expect the structure to be flattened out and much less fluxional.

The ‘weave pattern’ in 2D origami, where adjacent double helices tend to push apart and open up a significant gap between the helices away from the junctions, has been well known since the DNA origami method was originally devised, and is clearly visible in experimental microscopy images (3). It is also very apparent in the average structure depicted in Figure 2. This effect is not limited to DNA origami, but can also be clearly seen in, for example, arrays of DNA double-crossover tiles (91), including when modelled by oxDNA (57).

In Rothemund’s original paper, he suggested two possible reasons for this behaviour: firstly, electrostatic repulsion between the negatively charged helices; and secondly, that the detailed local structure around the junctions favours the helical arms to bend slightly away from each other (3). A third possible contribution is an entropic effect due to the increased conformational space available when adjacent double helices are not perfectly parallel. Here, we will see that all three of these effects play a role in the origin of the weave pattern for 2D origami in our oxDNA simulations.

**Figure 3.**
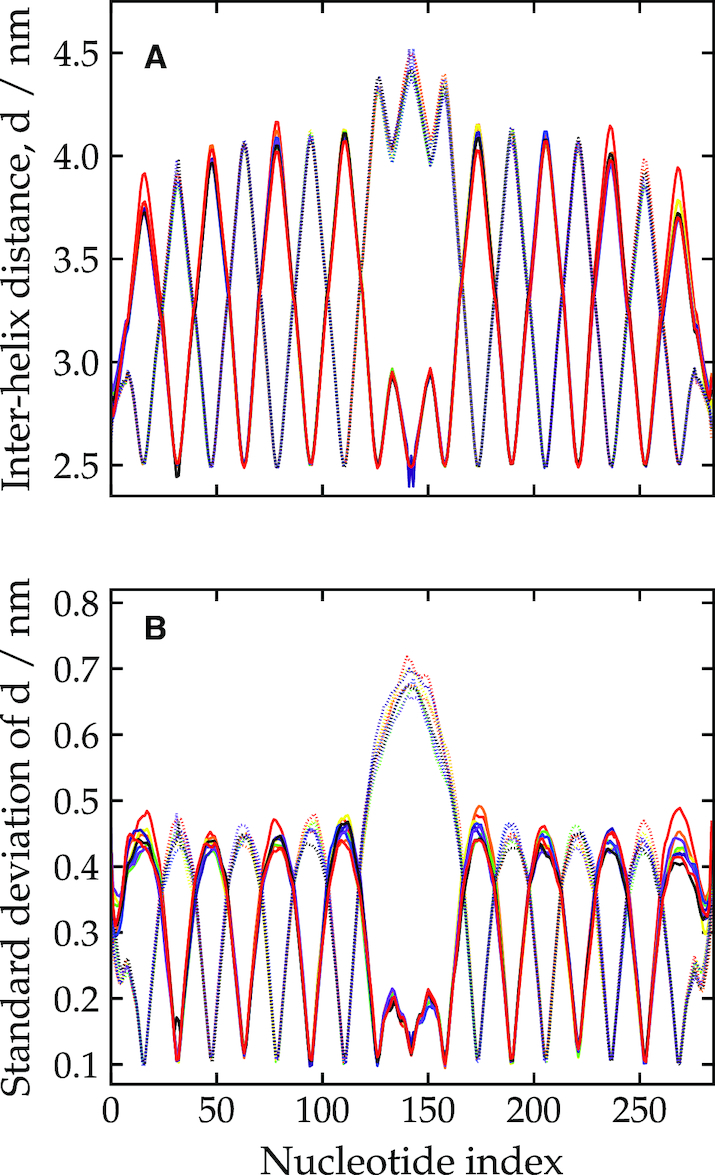
The weave pattern for the 2D tile at 300 K and [Na^+^] = 0.5 M, quantified by (**A**) the inter-helix distance and (**B**) the standard deviation in this distance as a function of base-pair index along the origami. Each line corresponds to a different pair of adjacent double helices on the origami. Some pairs have been omitted for clarity (see main text). The symmetry of the design is such that the pairs may be split into two groups: here one group is plotted with solid lines and one with dotted lines.

We quantify the weave pattern of the 2D tile by measuring the distance between the helix axes (defined as the midpoint between the bases for each base pair) for adjacent double helices. The results shown for the tile at a temperature of 300 K and [Na^+^] = 0.5 M are plotted in Figure 3. (Note, to simplify the appearance of the plot we omit some inter-helix distances. Namely, those involving the double helices at the top and bottom edges of the origami, as these are only constrained on one side and so exhibit slightly different behaviour; a few affected by branch migration which resulted in spurious results near the affected junction; and one affected by a partially melted staple that caused enhanced flexibility.) Because of the regular pattern of junction placement in the origami’s design (Supplementary Figure S2), there are two obvious groups into which the pairs of double helices can be divided. Each group exhibits a wave-like pattern with minima at the crossovers, where the double helices are brought closest together, and maxima away from the crossovers, normally at a position which is both midway between the junctions and where the adjacent pair of helices have a crossover. This pattern has a periodicity of about 32 base-pair steps, corresponding to the periodic junction placement in the origami.

In the middle of the plot (around base-pair index 150), a different pattern is evident. This is due to the presence of the origami’s seam (a series of junctions where the scaffold strand is exchanged), which runs along the middle of the tile. In this region, one group of double-helix pairs has a particularly large section without any junctions and so opens up to the largest extent here (3,92), as is also very clear from Figure 2; the modulations in the distance in the middle of this region reflect the presence of junctions on adjacent pairs of helices. By contrast, the other group of double-helix pairs has a shorter distance between junctions due to the extra scaffold crossovers, and opens up much less.

It is also interesting to note the ‘triangular wave’ character of the weave plots. The bending that creates the weave pattern is mostly localized at the junctions with the intervening sections basically straight. This is in part because the junctions are relatively flexible compared to the duplex sections between the junctions, which being only a small fraction of the persistence length (typically only 16 bp long compared to the 125 bp for the duplex persistence length for the model (38)) are very stiff. Furthermore, we can examine the fluctuations in the weave pattern, quantified in Figure 3(b) by the standard deviation in the inter-helix distances. The plot shows that the fluctuations, which are smallest at the junctions and largest at the midpoints between the junctions, are significantly smaller in magnitude than the variation in the interhelical distance due to the weave pattern itself. Thus, the junctions in the origami are very unlikely to adopt a configuration where the helices are straight with no weave (i.e. a value of θ near to zero) and have a static structural preference to be bent away from each other.

This is consistent with the picture that we obtained from the single Holliday junctions free-energy landscape, where for junctions that were near to anti-parallel, the free energy as a function of θ had a minimum at significantly positive θ (e.g. 4.5° for junctions in the range 160° < θ < 180°). We can also estimate the preferred value of θ at a junction in an origami from the weave pattern. Assuming a perfectly triangular wave form and taking 1.5 nm as a typical value of the difference in the interhelix distance between the maxima and minima of the weave pattern (Figure 3(a)) together with their 16 base-pair separation gives θ = 7.85°.

**Figure 4.**
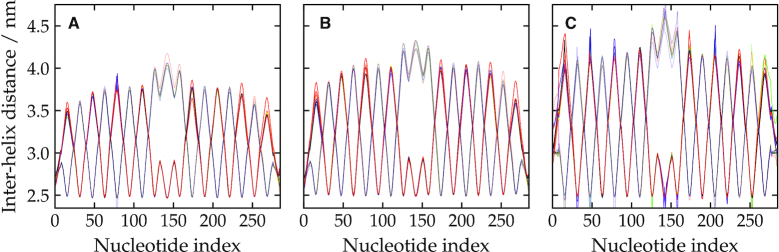
The weave pattern for the 2D tile (**A**) when the electrostatic term in the model is removed, and at [Na^+^] = 0.5 M and a temperature of (**B**) 270 K and (**C**) 330 K. The data is presented in an analogous way to Figure 3.

In order to investigate the effect of electrostatic repulsion, we repeated the simulations of the 2D tile with the electrostatic term in the potential removed. The result is shown in Figure 4A. We found that the weave pattern remained, albeit with a reduction in the magnitude of the oscillations by about 20%. This indicates that, although electrostatic repulsion enhances the weave pattern in oxDNA, it is not the sole cause. Experimental evidence for an increase in the inter-helical spacing for 3D origami as the ionic strength is decreased has been recently observed by SAXS of a 24-helix bundle (19) and by cryoEM of covalently-cross-linked origami (93).

Finally, we simulated the tile at different temperatures to obtain further insight into whether the weave pattern has a partly entropic, as well as energetic, origin. Figure 4B and C shows the weave pattern at 270 and 330 K. Together with the weave pattern at 300 K (Figure 3), the plots indicate that the magnitude of the oscillations characterising the weave pattern increases somewhat with increasing temperature. Thus, thermal fluctuations play a role in determining the magnitude of the weave pattern, with this entropic component favouring a more pronounced weave pattern. Again, this is consistent with the free-energy landscape in Figure 1C where the asymmetry of the minimum in the effective free-energy profile as a function of θ for anti-parallel junctions suggests that the average θ should increase as thermal fluctuations increase.

Are the interhelix distances predicted by oxDNA consistent with experiment? Taking the average between the maxima and minima of the triangular wave form gives an interhelix distance of about 3.25 Å at [Na^+^] = 0.5 M (Figure 3A) and 3.1 Å at the high ionic strength limit (Figure 4B), both at 300 K. Althous SAXS experiments have been performed on this origami design, the flexibility of the 2D origami meant that any features were too broad to back out an interhelix distance (21). However, these authors were able to provide more quantitative data for a 10-helix bundle tube with a similar pattern and spacing of junctions but without a seam. The best-fit diameter obtained by comparison to the predicted pattern for a bundle of perfectly straight helices implied an interhelix separation of ∼2.95 Å. These results were all at [Mg^2+^] = 12.5 mM, which (19) suggests is very close to the high ionic strength limit for interhelix separation. Given that the tube geometry and the lack of a seam is likely to reduce fluctuations somewhat compared to the 2D origami, our results are reasonably consistent with these values. Rothemund also provided an estimate of 3 Å for the interhelix separation based on AFM images of origamis adsorbed on a surface (again at [Mg^2+^] = 12.5 mM) (3). Note surface adsorption is also likely to suppress some of the fluctuations in the interhelix distance.

A second structural property that we see in 2D origami is what we term ‘corrugation’, where the origami displays a systematic, out-of-plane bending of the double helices, so that the junctions have a ϕ angle that is not exactly 180°, as would be the case for an origami with perfectly anti-parallel double helices. Due to the regularity of the origami design, this results in a wave-like pattern, albeit much smaller in magnitude than the weave pattern, on the surface of the origami that is visible for average-structure configurations, as shown in Figure 2D. That the free-energy for a free Holliday junction is a maximum for a perfectly anti-parallel junctions provides the driving force for this effect; for oxDNA this leads to a left-handed twist to the junctions.

Our approach to measure the corrugation is to follow how the orientations of the inter-helix vectors vary as one moves away from a junction. Specifically, for every such inter-helix vector we measure the angle between the inter-helix vector and the average inter-helix vector at the nearest junction between that pair of helices when projected onto the plane perpendicular to the average helix axis at that junction. The sign of the angle is determined from the sign of the scalar triple product of the two projected inter-helix vectors with the average helix axis, in order to distinguish clockwise and anticlockwise twisting. Thus, this measure quantifies the amount of twisting, in the plane perpendicular to the helix axis, between adjacent double helices near to junctions.

**Figure 5.**
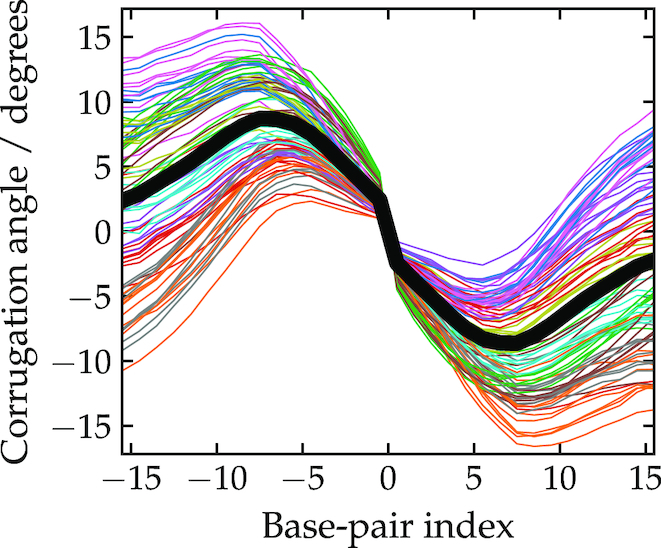
The corrugation pattern for the 2D tile. The angle between inter-helix vectors is is shown on the *y*-axis (see the main text for details of the definition). The *x*-axis shows the location of the inter-helix vector, in base-pair steps relative to the midpoint of the junction. Each line corresponds to a different junction, with the thick black line being an average over the data. Some junctions have been omitted for clarity (see main text).

The corrugation for the 2D tile as measured with this method is plotted in Figure 5. Note, we do not include data for all junctions, but only those that have the canonical pattern of neighbouring junctions; thus, we exclude the outermost junctions on the tile, and the junctions next to the scaffold seam as well as the seam itself. The plot shows the tendency for the double helices to come slightly out of the plane of the tile. The interhelix vectors are systematically rotated in one direction on one side of the junction (base pair index < 0) and in the opposite direction on the other (base pair index > 1). This corresponds to a ϕ angle of less than 180° for each junction, which is as expected from the properties of the free junction.

Although each junction shows qualitatively the same behaviour, there is clearly a wide variation in the curves for the base pairs furthest from the junction. Much of this is due to the method of twist compensation used in the origami design. Although overall the origami is roughly untwisted, this is achieved by having a mixture of interjunction double-helical sections that are over- and under-twisted (with 31 and 32 bp between junctions, respectively).

The general shape of the curves can be understood by considering the pattern of junctions in the origami. In order that adjacent junctions between a pair of double helices have the same twist, the curves must have (at least) one complete waveform every 31/32 bp. Thus, at the midpoint between the junctions the chiral twist angle must pass through zero. This position corresponds to the positions of junctions between adjacent helices and allows them also to have a left-handed chiral twist. Note that, unlike the weave pattern, the corrugation requires the maximum bending in between rather than at junctions, and so will be resisted by the bending stiffness of the duplex.

Although, to our knowledge, this corrugation effect has not been reported in any experimental studies of 2D origami, this is perhaps not surprising because the effect is small in magnitude and would tend to be reduced or removed when the structure is placed on a surface to be visualized, as is usually the case in experiments. The underlying cause of the corrugation is the tendency of the junctions to twist away from the perfectly anti-parallel conformation. Out-of-plane ‘distortions’ have also been noted in a series of planar ‘ring’ origami when modelled using CanDo (43), although in this case the junctions have a right-handed twist because the experimental Holliday junction conformation was set as the minimum of the harmonic junction twist potential.

### Structural properties of a 3D origami

**Figure 6.**
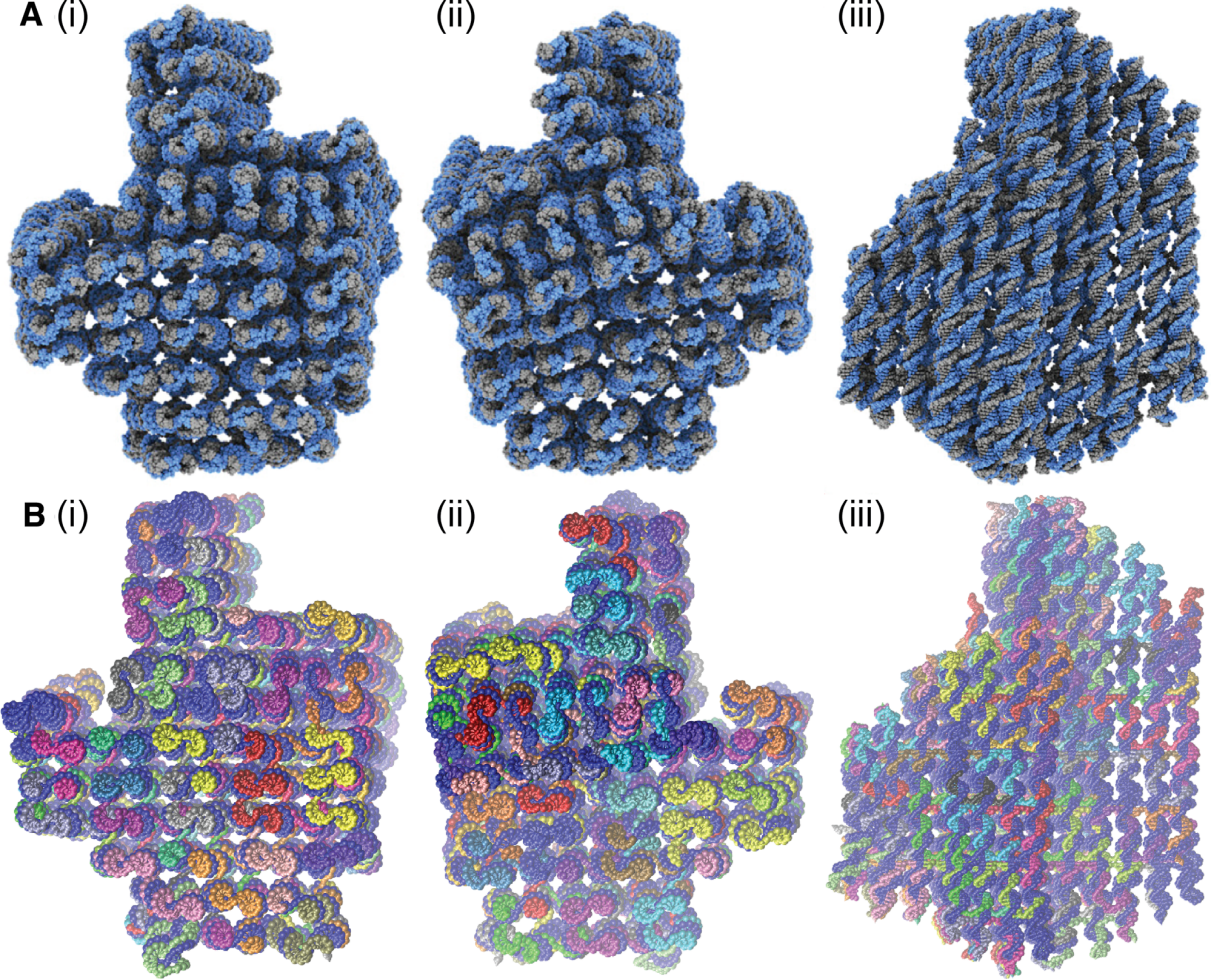
Three different views of the 3D origami structure determined by (**A**) fitting an atomistic model to cryo-EM data (reproduced from (24)) and (**B**) computing the average structure in simulations with the oxDNA model.

In this section, we further test the ability of oxDNA to accurately model DNA origami by making a detailed comparison to the 3D origami of (24) whose structure was characterised in detail by cryo-EM. The design uses a square lattice for the double helices, which means that the crossovers are spaced 32 bp steps apart, and we expect a slight global twist in the structure. The average structure computed from oxDNA simulations (see Supplementary Data for details of the averaging procedure) is compared to the experimentally-determined structure in Figure 6. By eye, the two structures appear very similar.

**Figure 7.**
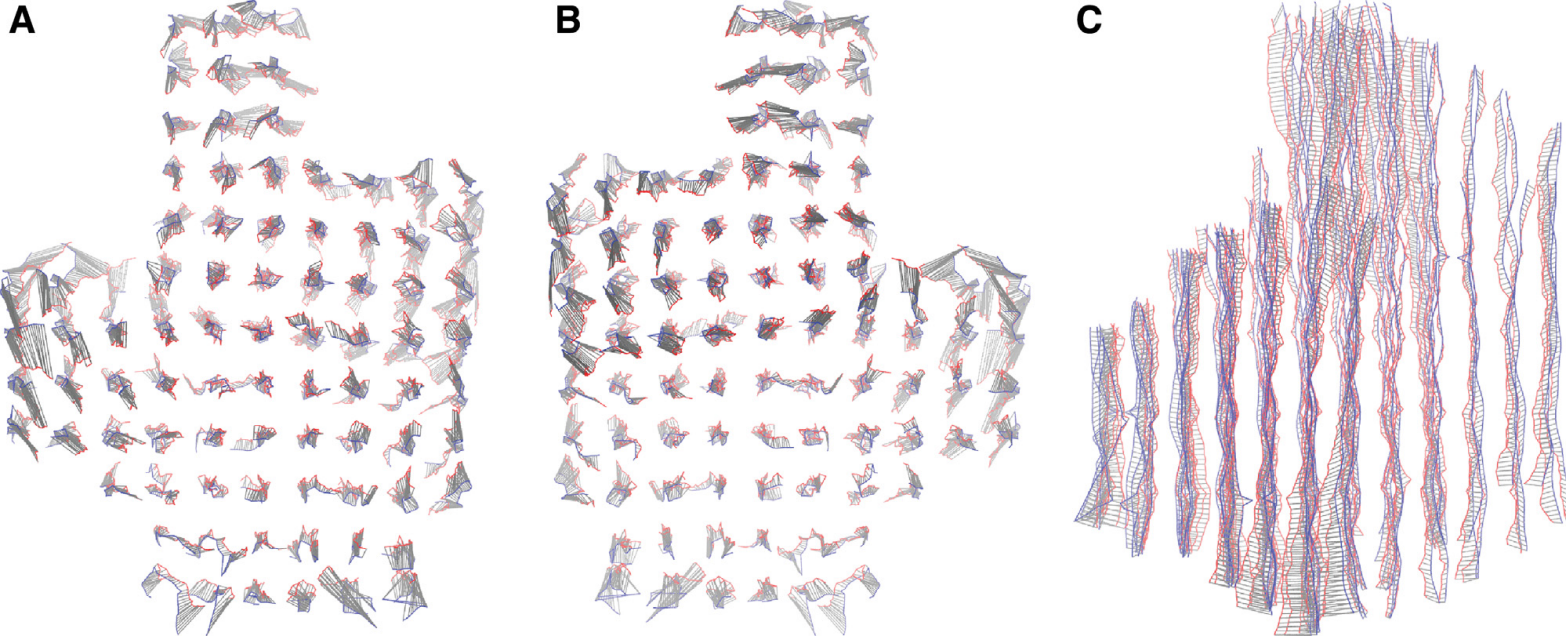
The aligned helix axes of the 3D origami that were used for the RMSD calculation (views as in Figure 6). The simulated structure is in blue, the experimental one is in red, and the grey lines show the displacement vectors.

To quantify this further we calculate the square root of the mean squared displacement (RMSD) between the simulated structure and the experimentally determined one. (Details of the RMSD calculation are given in the Supplementary Data.) We find that the RMSD is 0.84 nm, an excellent agreement with experiment. A graphical comparison is shown in Figure 7. From this figure, it is clear that the overall size of the cross-sectional lattice predicted by oxDNA is very close to the experimentally determined one, indicating that the magnitude of the weave pattern and the radius of the DNA double helix match experiment well. In addition, the overall twist of the structure is reproduced. The majority of the contribution to the RMSD is due to the double helices towards the outside of the structure, which are more clearly displaced from the experimentally determined structure. One potential reason for this disparity is that our average structure includes the effects of thermal fluctuations at room temperature, and it is not clear to what extent these fluctuations will be frozen in during the cryo-EM process. We also note that the estimated resolution of the cryo-EM characterization of the origami is reported as 0.97 nm at the core and 1.4 nm at the periphery, comparable to the RMSD we have found. For further comparison, fully-atomistic simulations of this origami were able to achieve an RMSD of 1.1 nm, which improved in the elastic-network guided simulations to 0.9 nm (34).

**Figure 8.**
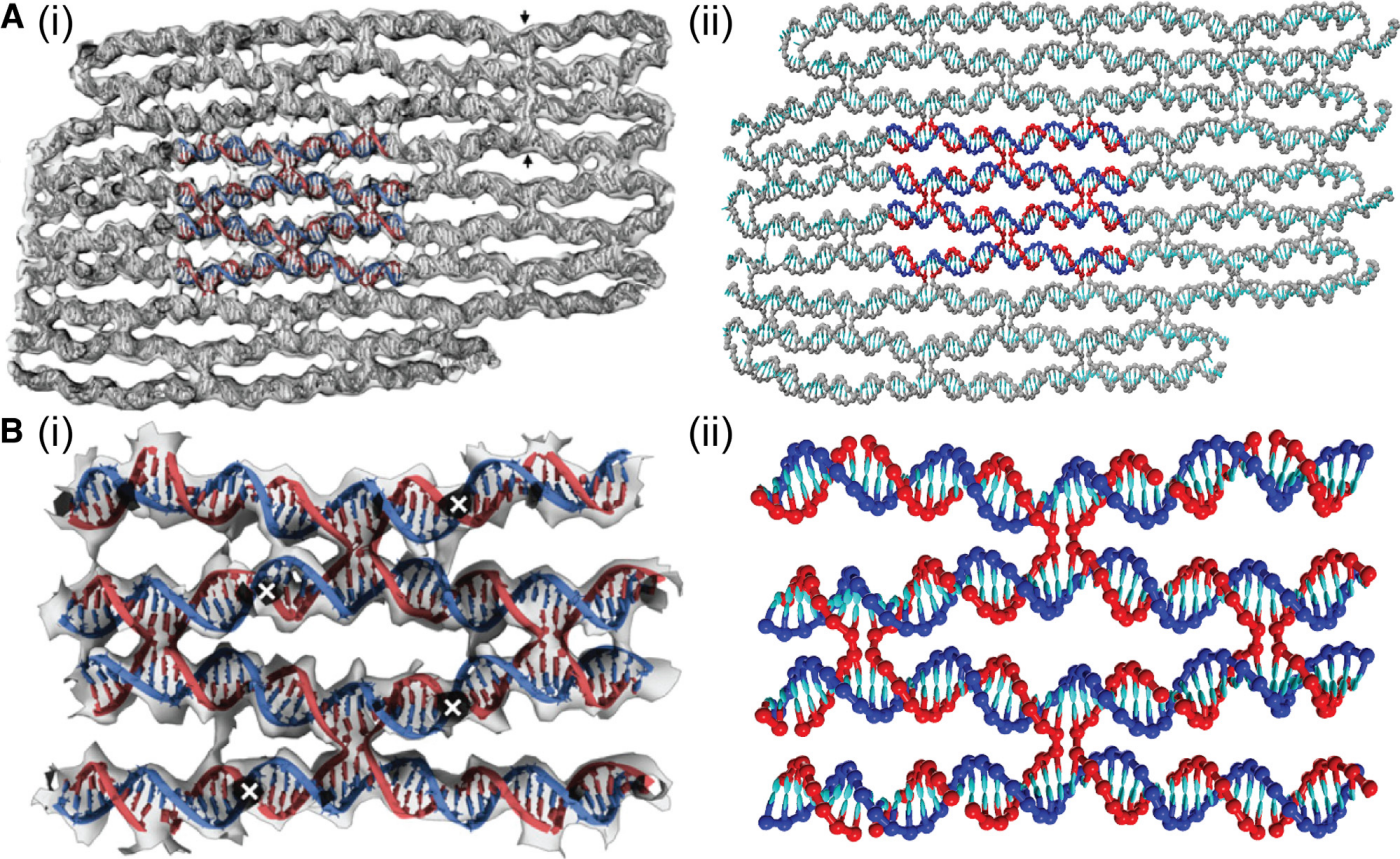
A comparison between experimental and oxDNA images for a typical plane from the 3D origami. (**A**) The whole plane and (**B**) a close-up of the highlighted region in (A). Images in (A)(i) and (B)(i) are reproduced from (24).

**Figure 9.**
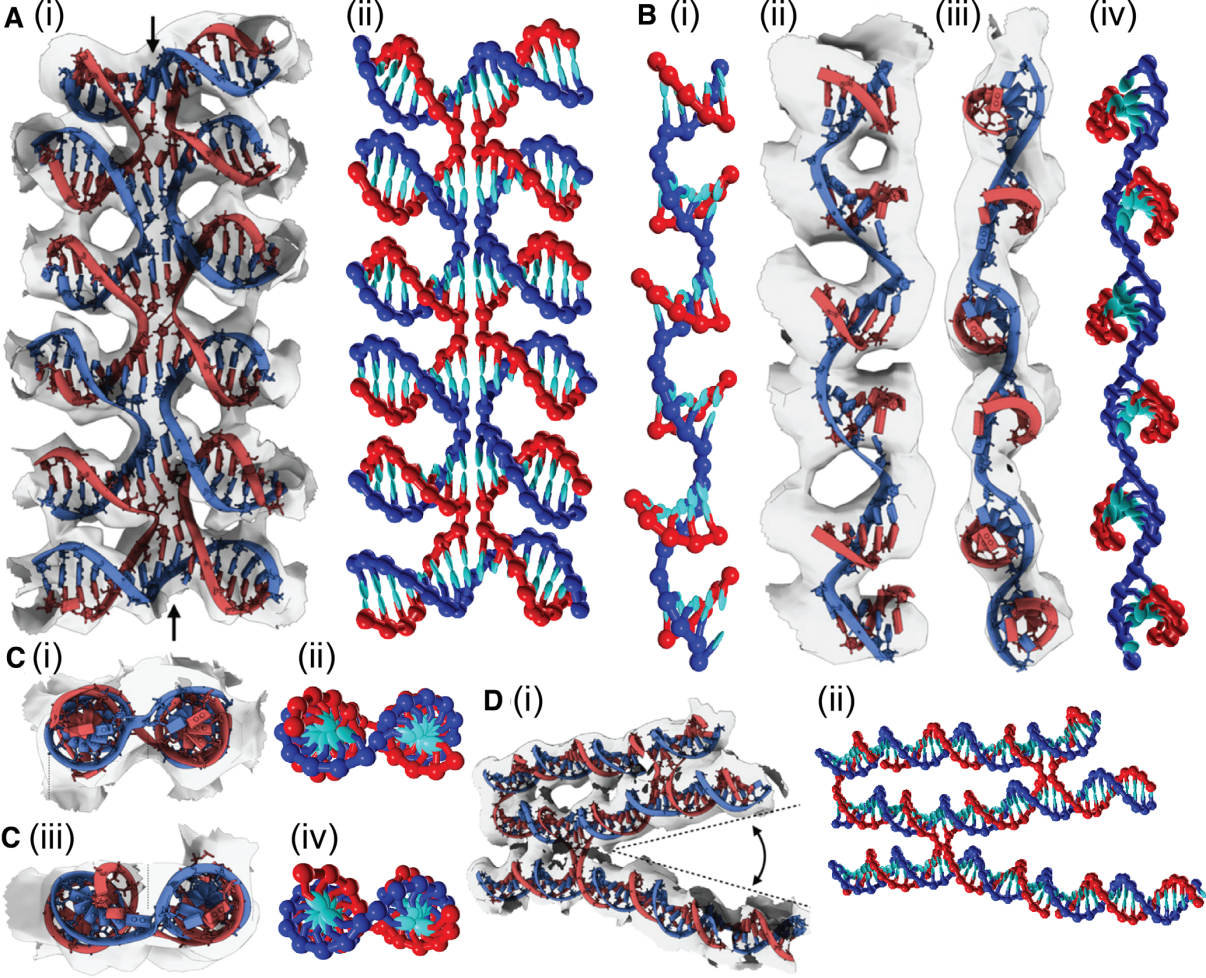
A comparison between experimental and oxDNA images for a variety of detailed motifs from the 3D origami. (**A**) Five aligned Holliday junctions that are held in place by coaxial stacking. (**B**) A section where the scaffold adopts a left-handed pseudo-helix associated with a set of five adjacent short domains. (**C**) A comparison of (i) and (ii) a typical Holliday junction in the origami with (iii) and (iv) one that is under twist stress due to there being one fewer base pair in an adjacent helix (often called a ‘deletion’). (**D**) An example of the greater splaying of helices that can occur at the ends of an origami due to a lack of constraints from other junctions. Images in (A)(i), (B)(ii) and (iii), (C)(i) and (iii), and (D)(i) are reproduced from (24).

Further evidence for the reliability of the oxDNA structure can be obtained by visual comparison of a variety of motifs from the origami. The examples, depicted in Figures 8 and 9, are the same as those chosen in (24). The similarity between the experimental and simulation images is very apparent. In particular, Figure 8 depicts a plane from the origami that, for the most part, has a similar pattern of junctions to the 2D origami, and therefore shows a very similar weave pattern, whereas Figure 9 provides a comparison of some more detailed motifs.

**Figure 10.**
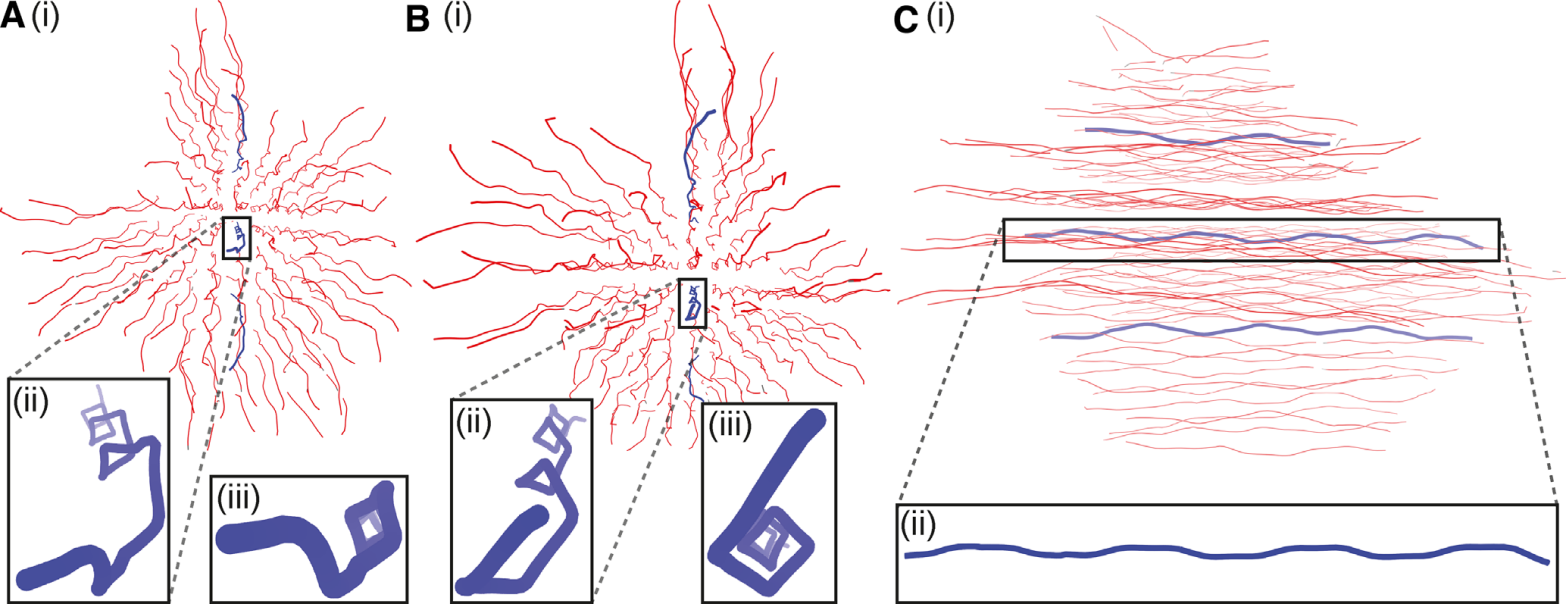
A representation of the 3D origami, where the red lines show the paths of the centres of the individual double helices from three different viewpoints. The pattern of junctions leads to these paths having a weak left-handed helicity, as highlighted by the blue paths in the insets.

One intriguing feature that we have identified is that the 3D origami’s double-helix axes trace out a left-handed helix with a period of ∼32 bp steps per turn, which corresponds to the spacing of junctions between each adjacent double helix pair in this design. This can be seen in Figure 10. We note that this weak feature is not clear in the experimental data perhaps because of the greater noise in the data.

This effect is mainly due to the ‘weave’ distortion, because unlike in the case of the 2D origami, the junctions do not lie in a single plane but in two orthogonal planes. In particular, as one moves along a double helix, it is drawn closer to each of its four neighbouring double helices in turn, as each junction is encountered. The junctions trace a left-handed helical path because they are separated by three-quarters of a pitch length along the right-handed double-helical sections. This path is also consistent with a left-handed chiral twist to each individual junction. Finally, that the helices possess a very weak toroidal writhe may be relevant when considering the precise effective pitch needed to generate an untwisted origami.

## CONCLUSION

In this paper, we have used the oxDNA coarse-grained model to characterize in detail some of the fundamental structural features of 2D and 3D DNA origami, and made a detailed comparison to the most detailed experimentally determined origami structure. In particular, we have shown that the weave pattern associated with the splaying of helices at the junctions has its origin in the basic structural properties of anti-parallel Holliday junctions, and is further enhanced by electrostatic repulsion and thermal fluctuations. For a 2D origami, we have also found a weaker out-of-plane corrugation associated with the slight left-handed twist of the Holliday junctions in the origami.

Our comparison to the 3D origami of (24), together with other recent studies of DNA origami using oxDNA (38,63–65,68,69), confirms the suitability of the oxDNA model for structural characterization of DNA origami. Structures that have been carefully characterised experimentally will give further opportunities to test and refine the structural predictions of the model, while for DNA origami that have only been visualized using low-resolution methods, oxDNA has the potential to provide more detailed structural insights, as has already been done for a number of examples (63,64,68,69). The model could also be used to pre-screen the properties of putative origami designs prior to experimental realization to aid the design process.

The capabilities of the oxDNA model should be seen as complementary to other computational strategies for origami structure prediction at different levels of detail. The particular advantages over more coarse-grained approaches potentially include explicit representations of excluded volume and base stacking, and realistic descriptions of single-stranded DNA and the breaking of base pairs, albeit, of course, at a greater computational cost. Furthermore, oxDNA provides a well-tested model for which a wide-range of biophysical properties of DNA are known to be accurately described. Examples of where these features will be particularly useful include origami with flexible components (64,69), origami under significant internal stresses, and origami where single-stranded components play a key functional role. The model also allows the loss of origami structure under thermal or external stresses (67) to be investigated. However, if a more atomically detailed view of an origami than is available through oxDNA is required, all-atom approaches are likely to be most appropriate.

## DATA AVAILABILITY

The simulation code is available online (https://dna.physics.ox.ac.uk.). Data associated with the simulations is available at the Oxford University Research Archive (DOI: 10.5287/bodleian:8gY5EnYYO).

## Supplementary Material

Supplementary Data
