# Supplementary material for: Coarse-grained modelling of the structural properties of DNA origami
Source: Nucleic Acids Res. 2019 Jan 3;47(3):1585–97. doi: 10.1093/nar/gky1304 (PMC6379721; doi:10.1093/nar/gky1304)
Supplement: Supplementary Data [file gky1304_supplemental_files.pdf]

Supplementary Data for "Coarse-grained modelling of the structural properties of DNA origami"

Benedict E. K. Snodin,[†] John S. Schreck,[‡] Flavio Romano,[¶] Ard A. Louis,[§] and Jonathan P. K. Doye[*,†]

Physical & Theoretical Chemistry Laboratory, Department of Chemistry, South Parks Road, Oxford, OX1 3QZ, United Kingdom, Department of Chemical Engineering, Columbia University, 500 W 120th Street, New York, NY 10027, USA, Dipartimento di Scienze Molecolari e Nanosistemi, Universit Ca' Foscari, Via Torino 155, 30172 Venezia Mestre, Italy, and Rudolf Peierls Centre for Theoretical Physics, University of Oxford, 1 Keble Road, Oxford, OX1 3NP, United Kingdom

E-mail: jonathan.doye@chem.ox.ac.uk

S1 Relaxation algorithm

Typically, the initial structures that are generated by converting a caDNAno file into a starting oxDNA configuration may locally be subject to very large forces due to overlaps

[*]To whom correspondence should be addressed

[†]Physical & Theoretical Chemistry Laboratory, Department of Chemistry, South Parks Road, Oxford, OX1 3QZ, United Kingdom

[‡]Department of Chemical Engineering, Columbia University, 500 W 120th Street, New York, NY 10027, USA

[¶]Dipartimento di Scienze Molecolari e Nanosistemi, Universit Ca' Foscari, Via Torino 155, 30172 Venezia Mestre, Italy

[§]Rudolf Peierls Centre for Theoretical Physics, University of Oxford, 1 Keble Road, Oxford, OX1 3NP, United Kingdom

or extended backbones. As such large forces are potentially problematic for a simulation (they may lead to an initial loss of structure or even cause the simulation to fail), we first relax configurations prior to them being simulated. The current recommended approach involves potentially two stages. The first stage is to apply a relatively short run of a simple steepest-descent minimization algorithm (with a fixed maximum step size) on the converted structure with the aim of resolving those problems that only require relatively small local structural changes (i.e. particle overlaps and some slightly extended backbones). During this minimization, we typically use a slightly modified version of the oxDNA potential. In particular, as the standard oxDNA backbone potential diverges beyond a certain separation, we replace the FENE backbone potential beyond a distance cutoff by one that smoothly evolves to a linear potential at large separation. Where this first step is by itself not sufficient to generate a suitable starting configuration, the second step is to run a normal simulation but with this modified backbone potential. This can allow any more large scale structural changes that are required for full relaxation. Details of how to convert a caDNAno design and subsequently relax it are also provided on the oxDNA website with an example of data files for the recommended approach being found in the `EXAMPLES/NEW_RELAX_PROCEDURE` folder of the oxDNA distribution.

For the origami considered in the current paper, the relaxation is a straightforward and rapid process because the changes in structure required to generate a good starting configuration are local and relatively minor, e.g. in response to the base-pair deletions in the 2D origami. For origami where larger, more global changes in structure are required (e.g. due to internal stresses that lead to global bending or twisting or due to an unphysically large separation of origami "blocks" in the caDNAno file) the process can be computationally more intensive, and in these cases that the second step of the relaxation approach can be run on a GPU is particularly helpful. However, if the conversion of the design file leads to a structure with entangled strands, the relaxation is unlikely to resolve this issue, because the explicit excluded volume in the oxDNA model prevents strands passing through each other. In such

cases, options include manual intervention to remove entanglements, or changing the layout of the caDNAno design. By contrast, such entanglements are typically not a problem for the CanDo relaxation algorithm because the CanDo model does not have any explicit excluded volume interactions;[1] this lack of excluded volume can however lead to structures involving overlaps for flexible origami where the structure is not mechanically fully constrained.

S2 Definitions of the ϕ and θ angles for a single Holliday junction

To compute ϕ and θ, we first define a plane with normal $\boldsymbol{n}$, given by

$$\boldsymbol{n} = \boldsymbol{A} \times \boldsymbol{B} + \boldsymbol{B} \times \boldsymbol{C} + \boldsymbol{C} \times \boldsymbol{D} + \boldsymbol{D} \times \boldsymbol{A}, \tag{S1}$$

where we define $\boldsymbol{A}$ as the normalised vector pointing along the helix axis of arm A. To be exact, $\boldsymbol{A}$ points from the base-base midpoint of the base pair closest to the crossover to the 4th closest base pair's base-base midpoint, as shown in Fig. S1(b). This means that $\boldsymbol{A}$ spans three base-pair steps. $\boldsymbol{B}$, $\boldsymbol{C}$ and $\boldsymbol{D}$ are defined in an analogous way. This definition is a compromise between not being too influenced by the thermal motion of one or two base pairs and not being too influenced by the bending fluctuations of the duplex arms far from the crossover.

In addition to this, we define an interhelix orientation vector $\boldsymbol{o}$ (Fig. S1(b))

$$\boldsymbol{o} = \frac{(\boldsymbol{b} + \boldsymbol{d})}{2} - \frac{(\boldsymbol{a} + \boldsymbol{c})}{2}, \tag{S2}$$

where $\boldsymbol{a}$ is the position vector of the base-base midpoint closest to the crossover of arm A, and likewise for $\boldsymbol{b}$, $\boldsymbol{c}$ and $\boldsymbol{d}$. $\boldsymbol{o}$ will allow us to define ϕ such that it runs from 0° to 360°, with a triple scalar product involving $\boldsymbol{o}$ being used to decide if ϕ is greater or less than 180°.

To compute ϕ, we define two twist angles, ϕ_1 and ϕ_2, which are a measure of the relative

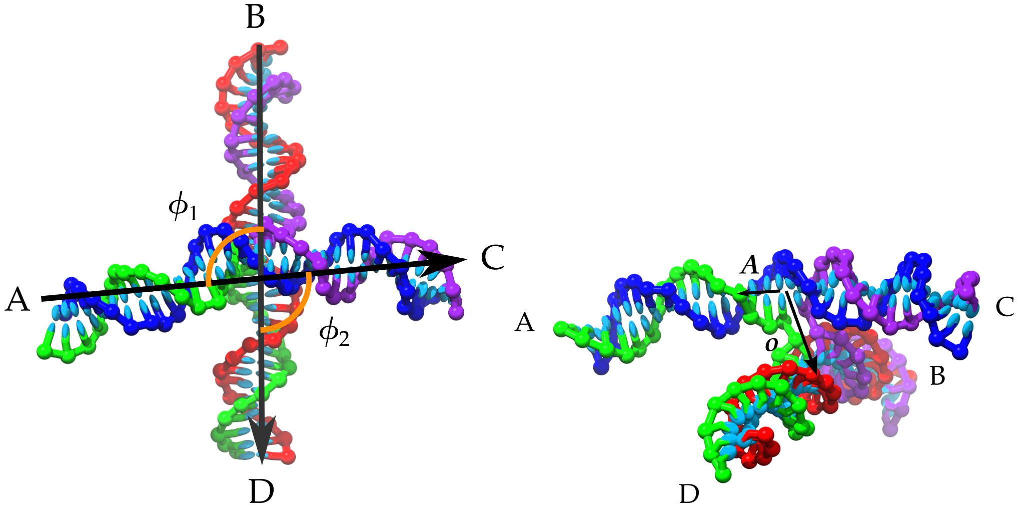

Figure S1: Two views of typical oxDNA configuration for a single Holliday junction to illustrate the definitions required to calculate θ and ϕ. (a) A top-view from perpendicular to the plane of the junction. Arrows point along the helix axes in the the 3' to 5' direction of the red and blue strands. The four junction arms are labelled A, B, C and D. The angles ϕ_1 and ϕ_2 (loosely speaking, the angles between arms A, B and C, D respectively) are shown, where $\phi = (\phi_1 + \phi_2)/2$. θ is the average angle between each arm and the plane of the junction. (b) A view from the side. The arm vector $\boldsymbol{A}$ and orientation vector $\boldsymbol{o}$ are shown.

twist of the Holliday junction arms around the axis of the crossover:

$$\phi_1 = \begin{cases} \arccos(\boldsymbol{A'} \cdot \boldsymbol{B'}) & \text{if } (\boldsymbol{A'} \times \boldsymbol{B'}) \cdot \boldsymbol{o} > 0 \\ 360^\circ - \arccos(\boldsymbol{A'} \cdot \boldsymbol{B'}) & \text{if } (\boldsymbol{A'} \times \boldsymbol{B'}) \cdot \boldsymbol{o} < 0 \end{cases},$$

$$\text{and} \tag{S3}$$

$$\phi_2 = \begin{cases} \arccos(\boldsymbol{C'} \cdot \boldsymbol{D'}) & \text{if } (\boldsymbol{C'} \times \boldsymbol{D'}) \cdot \boldsymbol{o} > 0 \\ 360^\circ - \arccos(\boldsymbol{C'} \cdot \boldsymbol{D'}) & \text{if } (\boldsymbol{C'} \times \boldsymbol{D'}) \cdot \boldsymbol{o} < 0 \end{cases},$$

where $\boldsymbol{A'} = (\boldsymbol{A} - (\hat{\boldsymbol{n}} \cdot \boldsymbol{A})\hat{\boldsymbol{n}})/|\boldsymbol{A} - (\hat{\boldsymbol{n}} \cdot \boldsymbol{A})\hat{\boldsymbol{n}}|$ is the normalised projection of the vector $\boldsymbol{A}$ towards the plane perpendicular to $\boldsymbol{n}$, with similar definitions for $\boldsymbol{B'}$, $\boldsymbol{C'}$ and $\boldsymbol{D'}$. ϕ is then defined as the average $\phi = (\phi_1 + \phi_2)/2$. Note that experimentally determined crystal structures have $\phi \approx 240^\circ$. (Often this value is quoted in terms of the deviation from an anti-parallel geometry, i.e. $\phi - 180 \approx 60^\circ$.)

To compute θ, we define angles θ_X, which are each related to the angle between the arm X and the plane of the Holliday junction:

$$\theta_A = \begin{cases} -|\arcsin(\boldsymbol{A}\cdot\hat{\boldsymbol{n}})| & \text{if } \boldsymbol{A}\cdot\boldsymbol{o} > 0 \\ |\arcsin(\boldsymbol{A}\cdot\hat{\boldsymbol{n}})| & \text{if } \boldsymbol{A}\cdot\boldsymbol{o} < 0 \end{cases},$$
$$\theta_B = \begin{cases} |\arcsin(\boldsymbol{B}\cdot\hat{\boldsymbol{n}})| & \text{if } \boldsymbol{B}\cdot\boldsymbol{o} > 0 \\ -|\arcsin(\boldsymbol{B}\cdot\hat{\boldsymbol{n}})| & \text{if } \boldsymbol{B}\cdot\boldsymbol{o} < 0 \end{cases},$$
$$\theta_C = \begin{cases} -|\arcsin(\boldsymbol{C}\cdot\hat{\boldsymbol{n}})| & \text{if } \boldsymbol{C}\cdot\boldsymbol{o} > 0 \\ |\arcsin(\boldsymbol{C}\cdot\hat{\boldsymbol{n}})| & \text{if } \boldsymbol{C}\cdot\boldsymbol{o} < 0 \end{cases}, \quad \text{(S4)}$$
$$\theta_D = \begin{cases} |\arcsin(\boldsymbol{D}\cdot\hat{\boldsymbol{n}})| & \text{if } \boldsymbol{D}\cdot\boldsymbol{o} > 0 \\ -|\arcsin(\boldsymbol{D}\cdot\hat{\boldsymbol{n}})| & \text{if } \boldsymbol{D}\cdot\boldsymbol{o} < 0 \end{cases},$$

This definition ensures that an arm pointing away from the plane through the centre of the junction that is normal to $\boldsymbol{n}$ has a positive angle and an arm pointing into the plane has a negative one. Note that by definition $\theta_A \equiv \theta_C$ and $\theta_B \equiv \theta_D$. This can be shown by considering each $\boldsymbol{X}\cdot\hat{\boldsymbol{n}}$ along with the definition of $\boldsymbol{n}$ given in Eq. S1. We define θ as the average of the θ_X angles: $\theta = (\theta_A + \theta_B)/2$.

S3 Holliday junction free-energy landscape

The sequences of the strands are given in Table SI. We windowed the simulations using a biasing potential defined as

$$V(\phi) = \begin{cases} k(\phi - (\phi_0 - \Delta))^2 & \text{if } \phi \le \phi_0 - \Delta, \\ 0 & \text{if } \phi_0 - \Delta < \phi \le \phi_0 + \Delta, \\ k(\phi - (\phi_0 + \Delta))^2 & \text{otherwise,} \end{cases} \quad \text{(S5)}$$

where k, ϕ_0 and Δ are constants. The relevant derivatives of this potential, requiring derivatives of ϕ with respect to the positions and orientations of the relevant nucleotides, were obtained so that the bias could be applied to MD simulations. As well as an unbiased simulation characterizing the region close to the free-energy minimum, additional simulations were performed for $\phi_0 = 140°$, $160°$, $180°$, $200°$ and $220°$ all with $\Delta = 40°$. For the biased simulations, only data from the flat region of the biasing potential, $\phi_0 - \Delta < \phi \leq \phi_0 + \Delta$, was used. The free-energy landscapes computed for each window were combined using the weighted histogram analysis method (WHAM).[2]

Table SI: The sequences used for the Holliday junction for which the free-energy landscape was computed.

strand	sequence
1	AGCAACTTGACGGTAAGAAGCAGAACTCTCCT
2	TCACTGCTAGGTGATCTTTGTTAAGGGTCTCA
3	TGAGACCCTTAACAAATTACCGTCAAGTTGCT
4	AGGAGAGTTCTGCTTCGATCACCTAGCAGTGA

It is possible for a free Holliday junction to form either one of two equivalent stacked isomers. The isomers are defined by which two strands are the exchanging strands, and which two stack through the junction. For these simulations, configurations where the Holliday junction had switched to the other, unintended isomer were discarded, as, although the behaviour of the two isomers should be equivalent, the definitions of θ and ϕ are isomer specific.

The nature of the isomer was determined by monitoring the distances between the four central base pairs at the centre of the junction. We denote these base pairs A, B, C and D, such that, in the intended isomer, each of the A bases usually stacks (or coaxially stacks) with each of the B bases, and likewise for the C and D bases. In addition, in the other isomer, the A bases will usually (coaxially) stack with the C bases, and the B bases with the D bases. We label the distance between the midpoints of the base pairs as d_{XY}, with d_{AB} denoting the distance between the A and B base-pair midpoints, for example. We then

define a configuration to correspond to the intended isomer when d_{AB} and d_{CD} are both shorter than either of d_{AC} and d_{BD}. This definition correctly identifies isomer flips, and was found to exclude only ∼1 in 1000 configurations, presumably due to extreme fluctuations, in trajectories where an isomer flip did not occur.

S4 Simulating 2D origami structure

The design of the "2D tile" used as the model system for the investigation of the weave pattern and other structural properties of 2D origami is shown in Fig. S2.

Both the 2D and 3D origami were simulated for a total of 10^9 steps with a time step of 0.005 (in the internal time units of the oxDNA model). Using the oxDNA units for energy, length and mass this maps onto a total time of $15\,\mu s$. In practice, the effective time is probably longer, as the time scale separation between diffusion times and the microscopic times (defined by the base units) are significantly reduced in simulations of such coarse-grained models.[3] This length of simulation is more than sufficient to sample the conformational fluctuations of the origami well. Configurations were saved every 10^6 steps and so measured properties were averages over 1000 configurations.

S5 Calculating an average structure

We used a relatively simple method to calculate an "average structure" of an origami from a simulation trajectory. This method has two steps: Firstly, local axes are computed for the origami for each configuration. This is done as follows: the x-axis is defined as the average direction of the vectors pointing along the double helix axes. For the 2D origami, a vector $\mathbf{v}$ is defined as the normal of the plane of the origami, which itself is given by the cross product between the average direction of the vectors pointing along the double helices and the average inter-helix vector between the top and bottom double helices. For the 3D origami, $\mathbf{v}$ is the average inter-helix vector between helices that are in the same row

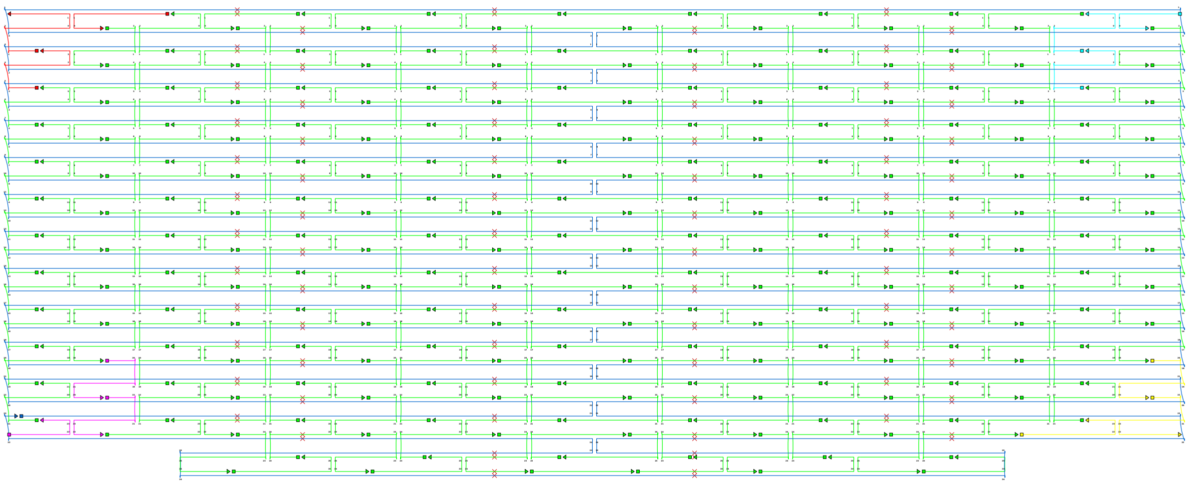

Figure S2: The caDNAno design for the 2D tile which was used as a model system for the investigation of the structural properties of 2D origami and has previously been investigated in Ref. 4. The scaffold strand is shown in blue, the staples are in green, and the red crosses indicate deletions (bases that have been removed from the design). Pairs of horizontal lines represent double helices and vertical lines represent junctions between them. Squares indicate the $5'$ end and triangles indicate the $3'$ end of each staple.

in the origami's caDNAno design. The z-axis is defined as the direction of the cross product between a vector pointing along the x-axis and $\mathbf{v}$, and the y-axis is defined as the direction of the cross product between a vector pointing along the z-axis and a vector pointing along the x-axis. Secondly, for each configuration the centre of mass is placed at the origin, and the positions and orientations of the nucleotides in this local coordinate system are computed. These are then added to the running total, which is normalised at the end of the procedure.

Thus, the average structure is an arithmetic mean of the coordinates and orientations of the nucleotides, in a reference frame which moves with the origami. This simple scheme is adequate for relatively stiff structures, but tends to give less reliable results for structures with significant flexibility, as the average positions of the nucleotides in these flexible parts will tend to shrink towards the centre.

S6 Comparison to experiment for the 3D origami structure

For the 3D origami, we quantify the level of agreement between the oxDNA model's average structure and the experimentally determined one by finding the RMSD between the two structures. The RMSD was calculated at the level of base pairs. That is, we measured one distance for each base pair in the structure, namely the distance between the base-pair midpoint in the simulated structure and that in the experimentally determined structure. We then took the average of the sum of the squares of these distances, and took the square root of the result.

The structures were superimposed on each other to facilitate the RMSD calculation as follows: First, the structures were translated so that the centres of mass were both at the origin. Then a vector pointing along a certain reference double helix was found for each structure, and the experimental structure was rotated so that these vectors were aligned. Using this as a starting point, a simple zero-temperature Monte Carlo algorithm was used to find the orientation of the experimental structure that minimised the RMSD. Trial moves were rotations with a random angle and rotation axis, and moves were accepted only if they lowered the RMSD. This procedure was repeated several times to check the result was robust and not just a local minimum.

References

1. Kim, D.-N.; Kilchherr, F.; Dietz, H.; Bathe, M. Quantitative prediction of 3D solution shape flexibility of nucleic acid nanostructures. *Nucl. Acids Res.* **2012**, *40*, 2862–2868.

2. Kumar, S.; Rosenberg, J. M.; Bouzida, D.; Swendsen, R. H.; Kollman, P. A. The weighted histogram analysis method for free-energy calculations on biomolecules. I. The method. *J. Comput. Chem.* **1992**, *13*, 1011–1021.

3. Doye, J. P. K.; Ouldridge, T. E.; Louis, A. A.; Romano, F.; Šulc, P.; Matek, C.; Snodin, B. E. K.; Rovigatti, L.; Schreck, J. S.; Harrison, R. M. *et al.* Coarse-graining DNA for simulations of DNA nanotechnology. *Phys. Chem. Chem. Phys.* **2013**, *15*, 20395–20414.

4. Baker, M. A. B.; Tuckwell, A. J.; Berengut, J. F.; Bath, J.; Benn, F.; Duff, A. P.; Whitten, A. E.; Dunn, K. E.; Hynson, R. M.; Turberfield, A. J. *et al.* Dimensions and global twist of single-layer DNA origami measured by small-angle X-ray scattering. *ACS Nano* **2018**, *12*, 5791–5799.
